# Advancing Systems Biology in the International Conference on Intelligent Biology and Medicine (ICIBM) 2015

**DOI:** 10.1186/s12918-016-0305-0

**Published:** 2016-08-26

**Authors:** Zhongming Zhao, Yunlong Liu, Yufei Huang, Kun Huang, Jianhua Ruan

**Affiliations:** 1 Center for Precision Health, School of Biomedical Informatics, The University of Texas Health Science Center at Houston, Houston, TX 77030 USA; 2 Department of Medical and Molecular Genetics, Indiana University School of Medicine, Indianapolis, IN 46202 USA; 3 Center for Computational Biology and Bioinformatics, Indiana University School of Medicine, Indianapolis, IN 46202 USA; 4 Department of Electrical and Computer Engineering, The University of Texas at San Antonio, San Antonio, TX 78249 USA; 5 Department of Biomedical Informatics, The Ohio State University, Columbus, OH 43210 USA; 6 Department of Computer Science, The University of Texas at San Antonio, San Antonio, TX 78249 USA

## Abstract

The 2015 International Conference on Intelligent Biology and Medicine (ICIBM 2015) was held on November 13–15, 2015 in Indianapolis, Indiana, USA. ICIBM 2015 included eight scientific sessions, three tutorial sessions, one poster session, and four keynote presentations that covered the frontier research in broad areas related to bioinformatics, systems biology, big data science, biomedical informatics, pharmacogenomics, and intelligent computing. Here, we present a summary of the 10 research articles that were selected from ICIBM 2015 and included in the supplement to *BMC Systems Biology*.

## Introduction

Since its inception in 2012, the International Conference on Intelligent Biology and Medicine (ICIBM) has hosted a variety of interdisciplinary educational events, including keynote lectures from world-renowned experts in bioinformatics, genomics, computational biology, bioengineering, and intelligent computing fields, educational and engaging tutorial and workshop sessions, poster presentations and discussions, and innovative research presentations in emerging areas like data science. ICIBM aims to provide a venue to promote interactions of trainees and junior scientists with established scientists and engineers so that their career can be developed at the right direction. ICIBM 2015, which was held on November 13–15, 2015 in Indianapolis, Indiana, USA, gained success again for promoting both education and research exchange. Specific conference program is described in our editorial to ICIBM supplemental issue to BMC Genomics [1]. Here, we briefly introduce the 10 research articles collected in this supplement to *BMC Systems Biology*. These articles covered research areas in machine learning approaches, models for big data, complex disease and drug prediction, drug-drug interactions and cellular networks, Boolean networks and computational tool for network analysis, among others. Each manuscript was reviewed by three or more reviewers and went through two rounds of reviews.

The first paper is to apply machine-learning approach to classify breast cancer patients. Breast cancer is a highly heterogeneous disease that is difficult to devise effective therapeutic strategies. In this study, Vural et al. uniquely used only somatic mutation profile data to identify and predict breast cancer subgroups [2]. The authors demonstrated that gene mutation profiles could be effectively used in unsupervised machine-learning methods to identify clinically distinguishable breast cancer subgroups. By using non-negative matrix factorization method, the authors identified three clinically distinct breast cancer groups. Furthermore, they developed a supervised classification model to predict the stage of the patients.

In the second paper, Xu and Wang developed a computational approach to characterize which and how human gut microbial metabolites may contribute to various aspects of Alzheimer’s disease (AD), a disease that is currently under extensive investigation [3]. They found metabolites were significantly associated with various aspects of AD, such as AD susceptibility, cognitive decline, biomarkers, age of onset, and the onset of AD, which provided evidence to support that human gut microbial metabolites might serve as an important mechanistic link between environmental exposure and AD symptoms. Furthermore, their pathway analysis revealed metabolite trimethylamine N-oxide (TMAO), a gut microbial metabolite of dietary meat and fat, providing insights into the mechanisms of dietary meat and fat on AD.

In the next paper, Li et al. digitalized a novel animal model for acute myeloid leukemia, which accounted for both leukemic and normal blood cells in the disease [4]. Specifically, the authors modeled multi-tissue hematopoietic dynamics in the leukemic body. The authors demonstrated that their computational model was valid and accurate, and that the functional depression of hematopoietic stem and progenitor cells under leukemia could be inferred by their model using the cell kinetics data as input only. Accordingly, their modeling is potentially capable of saving experimental costs, while effectively facilitating knowledge acquisition.

Cheng et al. developed a network-based framework to quantitatively examine cellular network heterogeneity and modularity in cancer [5]. Specifically, they constructed gene co-expressed protein interaction networks derived from large-scale RNA-Seq data across eight cancer types generated from The Cancer Genome Atlas (TCGA) project. They performed gene network entropy and balanced versus unbalanced motif analyses to investigate cellular network heterogeneity and modularity in tumor versus normal tissues, different stages of progression, and drug resistant versus sensitive cancer cell lines. The authors found that tumorigenesis could be characterized by a significant increase of gene network entropy in all of the eight cancer types examined. Furthermore, they showed that network entropy could be used to characterize tumor progression and anticancer drug responses. In addition, the authors provided potential network-level evidence that smoking might increase cancer cellular network heterogeneity and further contribute to tyrosine kinase inhibitor resistance. In summary, the authors demonstrated that network properties such as network entropy and unbalanced motifs associated with tumor initiation, progression, and anticancer drug responses, suggesting new potential network-based prognostic and predictive measure in cancer.

Predicting drug synergy using high-throughput expression profiles is an emerging topic in molecular cancer therapeutics development. Through integrative analyses of functional (modeled by gene sets) and gene-level data, Hsu et al. demonstrated that the similarity in functions or genes perturbed by two drugs was predictive of their synergistic effects based on the novel prediction scores measuring the synergy [6]. Assessed against a gold standard dataset derived by the DREAM consortium, the scores were shown to achieve better performance than previous computational methods. The results in this study are expected to facilitate the screening for putative synergistic drug combinations and accelerate the realization of precision cancer treatment.

Information about drug–drug interactions (DDIs) supported by scientific evidence is crucial for establishing computational knowledge bases for applications like pharmacovigilance. New reports of DDIs have been rapidly accumulating in the scientific literature; thus, text-mining techniques for automatic DDI extraction are critical. Zhang et al. proposed a novel approach for automated pharmacokinetic (PK) DDI detection that incorporates syntactic and semantic information into graph kernels, to address the problem of sparseness associated with syntactic-structural approaches [7]. The authors first developed a novel all-path graph kernel using shallow semantic representation of sentences. Then, they statistically integrated fine-granular semantic classes into the dependency and shallow semantic graphs. In the evaluation on the PK DDI corpus, their approach significantly outperformed the original all-path graph kernel, which is based on dependency structure.

In the next paper, Du et al. introduced a novel sparse canonical correlation analysis (SCCA) model using the Graph OSCAR constraint [8]. The model was developed for identifying bi-multivariate imaging genetic associations while encouraging highly correlated features to be selected together. Because the model does not require the pre-definition of the sign of the sample correlation, it could reduce the estimation bias. Furthermore, it could combine those highly correlated features together regardless of whether they are positively or negatively correlated. The authors showed that the performance of the model would be promising based on both simulated and real data, and they identified a strong relationship between the *APOE* SNP rs429358 and the amyloid burden measure in the frontal region.

We have witnessed numerous high-throughput “omics” data generated during the past decade. While the data is large, it is often complicated. The biomedical scientists have been hindered from fully studying and uncovering disease associated networks by the lack of efficient computational tools. Wan et al. introduced an open source R package to help learn the network structure from data acquired from various high-throughput genomics technologies [9]. Their tool allows the data to be modeled using the native distribution instead of normalizing the data to follow Gaussian distribution, as most other statistical models require. In addition, the parallelization of their algorithms provides an efficient tool for computing large-scale networks.

Liu et al. explored controllability of a Boolean network based on the transition matrix and time transition diagram [10]. In their study, the authors determined the necessary and sufficient condition for a controllable Boolean network and mapped this requirement in transition matrix to real Boolean functions and structure property of a network. They found six simplest forms of controllable 2-node Boolean networks and explored the consistency of transition matrices while extending these six forms to controllable networks with more nodes. Next, the authors proposed the first state feedback control strategy to drive the network based on the status of all nodes in the network. In a case study, the authors applied the reachability condition to the major switch of P53 pathway to predict the progression of the pathway and validate the prediction with published experimental results. The control strategy reported in their paper allows investigators to apply real time control to drive Boolean networks, a feature having missed in the current control strategy for Boolean networks.

In the last paper, Wu et al. applied an integrative gene co-expression analysis approach to identify a novel, three-transcription factor based prognostic signature [11]. The signature could cluster the glioma into three molecular subtypes with significantly different transcriptomic patterns and clinical outcomes. The authors explored the glioma molecular mechanisms at the transcriptional regulation level. They suggested potential drug targets for different glioma molecular subtypes. This integrative approach is extendable to other cancer studies for detecting biomarkers in both diagnosis and pathogenesis.
